# Incidence des infections du site opératoire en Afrique sub-saharienne: revue systématique et méta-analyse

**DOI:** 10.11604/pamj.2016.24.171.9754

**Published:** 2016-06-29

**Authors:** Ngaroua, Joseph Eloundou Ngah, Thomas Bénet, Yaouba Djibrilla

**Affiliations:** 1 Service de Chirurgie, Hôpital Régional de Ngaoundéré, Cameroun; 2 Département des Sciences Biomédicales, Université de Ngaoundéré, Cameroun; 3 Neurochirurgie, Faculté de Médecine et des Sciences Biomédicales, Université de Yaoundé I, Cameroun; 4 Service d’Hygiène, Epidémiologie et Prévention, Hôpital Edouard Herriot, Hospices Civils de Lyon, France; 5 Laboratoire des Pathogènes Emergents, Fondation Mérieux, Centre International de Recherche en Infectiologie (CIRI), Inserm U1111, CNRS UMR5308, ENS de Lyon, UCBL1, Lyon, France

**Keywords:** Infections du site opératoire, incidence, facteurs de risque, Afrique Sub-saharienne, Surgical site infections, Incidence, risk factors, Sub-Saharan Africa

## Abstract

**Introduction:**

Les Infections du Sites Opératoire (ISO) sont à l’origine de morbi-mortalité et des dépenses supplémentaires en santé. Les pays en développement en sont les plus touchés. L’objectif était d’estimer l’incidence poolée des ISO en Afrique Sub-saharienne et décrire ses principaux facteurs de risque.

**Méthodes:**

Une revue systématique et une méta-analyse ont été effectuées à partir des bases de données de l’Organisation Mondiale de la Santé pour la Région Afrique, de PubMed et par recherche standard afin de sélectionner des articles électroniquespubliés entre 2006 et 2015. Seuls les articlestraitants de l’incidence et desfacteurs de risque des ISOdans les pays del’Afrique subsaharienneétaient retenus.

**Résultats:**

Sur 95 articles trouvés, 11 ont répondu aux critères d’inclusion. Seulement 9 pays sur les 45 y ont contribués avec une grandereprésentation du Nigéria (5 articles sur 11). L’incidence des ISO variaient de 6,8% à 26% avec une prédominance en chirurgie générale. L’incidence poolée des ISO était de 14.8% (IC à 95%: 15,5-16,2%), avec une importante hétérogénéité selon la spécialité et le mode de surveillance. Les facteurs de risque les plus citésétaient la longue durée d’intervention et la classe de contamination d’Altemeir 3 et 4. Les autres facteurs concernaient l’environnement hospitalier, les pratiques de soins inadéquats et les pathologies sous-jacentes.

**Conclusion:**

L’incidence des ISO est élevée en Afrique subsaharienne, des études dans cette région pourrait améliorer la connaissance, laprévention et la maitrise deces multiples facteurs de risques.

## Abstract (en)

**Introduction:**

Surgical Site Infections (SSI) cause morbi-mortality and additional healthcare expenditures. Developing countries are the most affected. The objective was to estimate the pooled incidence of SSI in Sub-Saharan Africa and describe its major risk factors.

**Methods:**

Systematic review and meta-analysis were conducted using the databases of the World Health Organization Regional Office for Africa, PubMed and standard search to select electronic articles published between 2006 and 2015. Only articles investigating SSI impact and risk factors in Sub-Saharan African countries were retained.

**Results:**

Out of 95 articles found, 11 met the inclusion criteria. Only 9 countries out of 45 have contributed, with a huge amount of information coming from Nigeria (5 articles out of 11). The impact of SSI ranged from 6.8% to 26% with predominance in general surgery. The pooled incidence of SSI was 14.8% (95% CI: 15,5-16,2%) with significant heterogeneity according to the specialty and the method of monitoring. Most cited risk factors were long procedure length and categories 3 and 4 of Altemeier contamination class. Other factors included hospital environment, inadequate care practices and underlying pathologies.

**Conclusion:**

SSI incidence is high in Sub-Saharan Africa. Studies in this area could improve knowledge, prevention and control of these multiple risk factors.

## Introduction

Les Infections du Site Opératoire (ISO) sont l’une des principales causes de mortalité et de morbidité en chirurgie. Sa survenuelimite le bénéfice potentiel des interventions chirurgicales et multiplie par trois le coût d’hospitalisation [1]. Elles sont ainsi à l’origine d’énorme dépense en santé. En Afrique subsaharien, son incidence seraitélevée etdes facteurs économiques et sociaux constitueraient des grandes barrières à la prévention deces infections [2, 3]. A notre connaissance aucune étude n’a spécifiquement estimée l’incidence poolée des ISO et recherchée ses facteurs de risque en Afrique sub-saharienne. L’objectif de ce travail est d’estimer l’incidence poolée des ISO en Afrique Sub-saharienne et de décrire ses principaux facteurs de risque dans une revue systématique et méta-analyse.

## Méthodes

Une recherche systématique et avancée sans restriction de langue à partir des mots cléssur la thématique a été réalisée dans les Bases de données suivantes: Index Medicus Santé Africaine de l’Organisation Mondiale de la Santé (AFROLYB, AIM, GHL Global Heath Library); Pubmed; Google Scholar et une recherche standard à l’aide des robots de recherche. Elle concernait les titres des articles, les résumés, les rapports, les mémoires et tout autre présentation électronique, sans restriction de type de format et d’année concernant l’Afrique subsaharienne. L’Afrique subsaharienne est entendue comme la partie du continent africain située au sud du Sahara, séparée écologiquement, culturellement, ethniquement, des pays du nord par le climat rude du plus vaste désert du monde et regroupant 48 pays. Les mots clés recherchés étaient: « Infections du Site Opératoire », « infections de plaies opératoires », « facteurs de risque », « étude prospective », « Afrique subsaharienne ». Les séparateurs logiques ET (AND) et OU (OR) ont été utilisés à cet effet pour associer ces mots.

Les itemsde recherché utilisés étaient: **((tw:(surgical site infection)) OR (tw:(surgical wound infections)) AND (tw:(risk factors)) OR (tw:(factor of risk)) AND (tw:(Africa))); (tw:(surgical wound infection risk factors Africa) AND (instance:"ghl")); (tw:(surgical site infection risk factors africa) AND (instance:"ghl")); Pubmed (((((surgical site infection) OR surgical wound infection) AND risk factors) AND Africa) OR sub-Saharan)**.

Les critères d’inclusion des articles étaient: #1) étudeprospective, et #2) menée dans tout type de structure sanitaire de l’Afrique Subsaharien entre 2006 et 2015, et #3) dont les sujets sont des êtres humains, et #4) quelle que soit la discipline chirurgicale et #5) comprenant des informations descriptives sur le ISO et ses principaux facteurs de risque. Les pays Africains n’appartenant pas à la partie subsaharienne ne sont pas retenus. Les informations suivantes ont été recueillies pour chaque étude: référence de l’étude, année de publication, nombre d’ISO, population surveillée, suivi actif des patients, données microbiologiques et spécialité chirurgicale. Les résultats sont présentés selon la pertinence des recherches, des auteurs, des types d’études et des critères d’inclusions. L’incidence poolée des ISO a été estimée par la méthode de l’inverse de la variance [4–6] sous Stata 11.0 (Stat Corp), globalement puis stratifiées sur le type de spécialité chirurgicale et les modalités de surveillance (données microbiologiques ou non).

## Résultats

**Figure 1:**
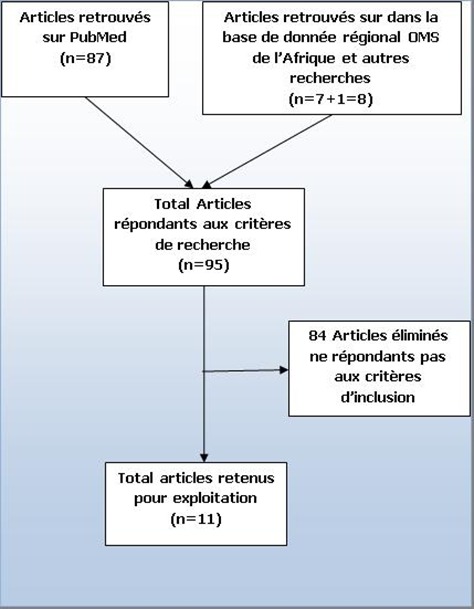
Schéma simplifiés de sélection des articles sur les ISO en Afrique Subsaharien entre 2006 et 2015

**Figure 2:**
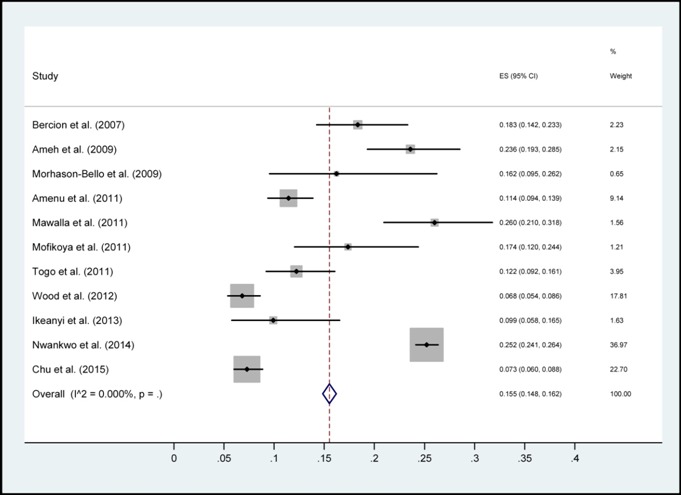
Incidence poolée des infections du site opératoire

**Figure 3:**
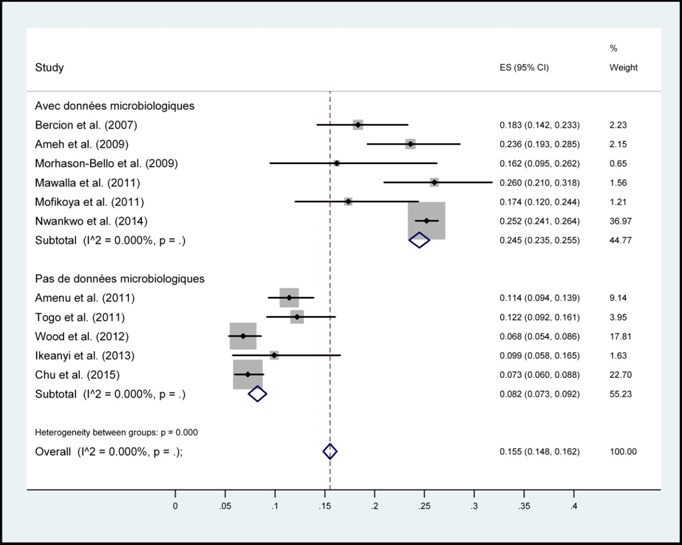
Incidence poolée des infections du site opératoire en Afrique subsaharienne, stratifiée selon la présence de données microbiologiques

**Figure 4:**
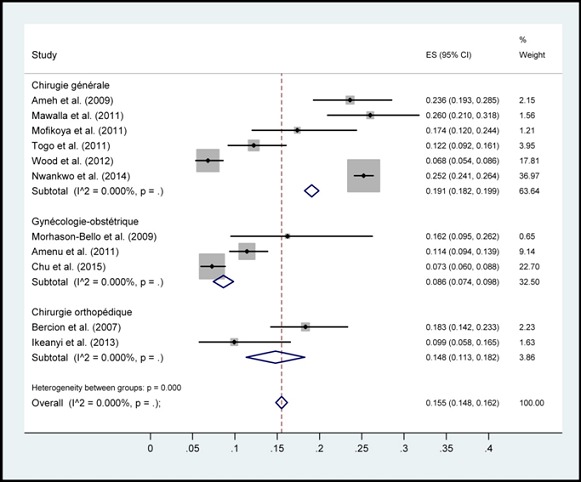
Incidence poolée des infections du site opératoire en Afrique subsaharienne, stratifiée selon la spécialité chirurgicale

Au total, sur 95 articles indexés, 11ont répondu aux critères d’inclusion (Figure 1) car les autres n’étaient pas de types prospectifs et les facteurs de risque n’étaient pas mis en évidence. Ces articles sont classés par auteurs, date, taille d’échantillon, pays, et spécialités chirurgicales (Tableau 1). Ensuite, les pays cités sont repartis selon leur nombre de publication sur les ISO. Ces pays étaient: la RépubliqueCentrafricaine, le Burundi, la Sierra Leone, le Mali, la Tanzanie, le Kenya, la République Démocratique du Congo, l’Ethiopie et le Nigéria. Chacun de ces pays avait une seule publication que nous avons sélectionnée, excepté le Nigéria qui en avait cinq articles. L’incidencepoolée des infections du site opératoire était de 14.8% (intervalle de confiance à 95%: 15.5-16.2%) (Figure 2). Cette incidence stratifiée selon la présence de données microbiologiques et selon la spécialité chirurgicaleont permis d’énumérerles principaux facteurs de risque des ISO (Figure 3, Figure 4). En effets, les facteurs de risquescités par les différents articles étaient :la longue durée d’intervention chirurgicale (6 fois), le stade de contamination élevé chez le patient ou Classe d’Altemeir 3 et 4(4 fois); l’anémie (3 fois); la présence de drain (2 fois), le défaut de préparation des malades (4 fois) et un longséjourpréopératoire. Les autres facteurs concernaient l’environnement hospitalier, les pratiques de soins inadéquats, l’âge extrême, la malnutrition, l’absence de consultation prénatale, les pathologies maternelles, le niveau d’instruction faible et les pathologies sous-jacentes.

**Tableau 1:** Synthèses des articles sélectionnés pour l'étude

Auteurs	Pays	échantillon	incidence des ISO	germes isolé?	Spécialité chirurgicale
**Ahme et *al. 2009***	Nigeria [9]	322+	23,6%	Oui	Générale
**Amenu et *al. 2011***	Ethiopie [14]	770++	11,4%	Non	Obstétrique
**Bercion et *al. 2007***	RCA [7]	278+++	18%	Oui	Orthopédie
**Chu et *al. 2015***	RDC, Sierra Leone, Burundi [8]	1276++	7,3%	Non	Obstétrique
**Togo et *al. 2011***	mali [15]	352+++	12,2%	Non	Générale
**Ikeanyi et *al. 2013***	Nigeria [10]	121+++	9,9%	Non	Orthopédie
**Morhason et *al.* 2009**	Nigeria [11]	75++	16,2%	Oui	Obstétrique
**Mawalla et *al. 2011***	Tanzanie [17]	250+++	26%	Oui	Générale
**Nwankwo et *al.* 2014**	Nigeria [12]	5800+++	25,2%	Oui	Générale
**Mofikoya et *al.* 2011**	Nigeria [13]	144+++	17,4%	Oui	Abdominale
**Wood et *al. 2012***	Kenya [16]	940+++	6,8%	Non	Générale

+ sujets enfants uniquement

++ sujets femmes uniquement

+++ sujets sans distinction

## Discussion

### Caractéristiques des études

Entre 2006 et 2015, 11 articles répondants aux critères d’inclusion ont été retenus après dépouillement et relecture des titres et résumés de 95 articles indexés (Figure 1). Seulement 9pays sur les 48 que compte l’Afrique Subsaharien étaientreprésentés. Il s’agit de: République Centrafricaine [7], Congo [8], Nigeria [9–13], Ethiopie [14], Mali [15], Sierra Léone [8], Kenya [16], Burundi [8] et Tanzanie [17]; avec une forte représentation du Nigeria (Tableau 1). Il est possible que des études non publiées aient été menées dans cette région durant cette période d’étude, mais nous notons que les résultats pourraient être approximativement les mêmes. Ces études concernaient pour la plupart la chirurgie générale (42%), obstétricale (33%), orthopédique (17%) et abdominale (8%). Certains auteurs se sont intéressés à des sujets spécifiques tels que les enfants (1 article), les parturientes (2 articles), alors que d’autres avaient inclus tout patients admis pour intervention chirurgicale sans spécificité du genre. Les germes étaient isolés dans 6études sur 11 (54,54%). Les 11 articles publiés que nous avons retrouvés en Afrique Subsaharienne entre 2006 et 2015 par cette revue systématique sont ceux qui ont étudié spécifiquement et de manière prospective les ISO. Notons que dans la plupart deces pays, le système de santé est organisé autour d’une structure pyramidale avec à la base les centres de santé, les hôpitaux de district au niveau intermédiaire et les hôpitaux de référence préfectoraux, régionaux ou nationaux au sommet de la pyramide. L’hôpital est un lieu où l’on prodigue des soins médicaux et chirurgicaux. Les services publics cohabitent généralement avec le système de Santé privé retrouvé majoritairement dans les grandes villes africaines. Il s’agit des cliniques bien équipées avec un personnel médical de qualité. Ces structures sont payantes et ne sont accessibles que pour les populations les plus favorisées [18]. De nombreux facteurs contribuent à l’absence de progrès: un faible niveau de gouvernance et de responsabilisation, l’instabilité politique, les catastrophes naturelles, les infrastructures sous-développées, les faiblesses du système de santé et le manque d’harmonisation et d’alignement de l’aide [19, 20].

### Incidence des ISO

Les études recensées montrent un taux élevé des ISO dans les hôpitaux allant de 6,80% à 26% en Afrique Subsaharienne (Tableau 1). Elle était plus élevée au Nigeria avec une incidence de 25,2% (n=5800). L’incidence poolée des ISO était de 14.8% (intervalle de confiance (IC) à 95%: 15.5-16.2%)tous pays confondus(Figure 2). Une importante hétérogénéité de l’incidence était observée selon le type de spécialité avec une incidence des ISO de 19,1% (IC 95%: 18,2-19,9%) en chirurgie générale et viscérale, de 14,8% (IC 95%: 11,3-18,3%) en orthopédie et de 8,6% (IC 95%: 7,4-9,8%) en gynécologie obstétrique. L’incidence des ISO était supérieure lorsque des données microbiologiques de surveillance étaient disponibles24, 5% (IC 95%: 23,5-25,5%) et 8,2% (7,3-9,2%) en l’absence de données microbiologiques (Figure 3, Figure 4). Ceci est à l’origine des surcoûts liés à la prise en charge postopératoire, à une morbi-mortalité élevée et peut altérer l’image des formations hospitalières. A l’opposé, les progrès faits en matière de surveillance des infections nosocomiales dans les pays développés ont permis de réduire son incidence. On retrouve par exemple en France des taux de 1,16% (en 2007 et 2008) à 1 % (en 2009 et 2010) et continue à baisser grâce à la surveillance continue [21]. Mais aussi en Afrique du Nord, des taux plus bas sont retrouvés; par exemple une incidence de 5,2% au Maroc [22]. Même si des études similaires ont généralisés les infections nosocomiales en chirurgie sans spécificité sur les ISO [23, 24], son incidence est toujours le plus élevé. L’hétérogénéité de l’incidence selon la spécialité est expliquée par des niveaux de risque et de propreté différente avec une incidence supérieure en chirurgie générale et digestive qui est plus fréquemment une chirurgie contaminée (Figure 4). L’hétérogénéité des taux d’ISO selon la présence de données microbiologiques ou non souligne l’apport des informations bactériologiques dans la prise en charge du patient mais aussi pour l’estimation précise de l’incidence. Une méta-analyse similaire en chine sur 84 articles sélectionnés avait trouvée une incidence moyenne des ISO de 5% (95% CI: 3.1-5.8) entre 2001 et 2012. La stratification par spécialité chirurgicale a montrée une incidence élevée en chirurgie abdominale (8.3%, 95% CI: 6.5-10.0) [25].

### Facteurs de risque des ISO

Les principaux facteurs de risque des ISO concernaient autant les soignants que des patients. La longue durée d’intervention chirurgicale (6 fois), le stade de contamination élevé chez le patient ou Classe d’Altemeir 3 et 4(4 fois); l’anémie (3 fois); la présence de drain (2 fois), le défaut de préparation des malades (4 fois) et un longséjourpréopératoire. Les autres facteurs concernaient l’environnement hospitalier, les pratiques de soins inadéquats, l’âge extrême, la malnutrition, l’absence de consultation prénatale, les pathologies maternelles, le niveau d’instruction faible et les pathologies sous-jacentes. La présence de drain en effet fait l’objet de controverse dans son implication dans les ISO. Une revuesystématique n’a pas pu conclure sa relation avec la survenue des ces infections [26]. Une étude similaire international sur les facteurs de risqué des ISO a identifiée les facteurs suivante :la co-morbidité, l’âge extrême, l’indice de risque élevé et la complexité de l’intervention. Par ailleurs, le diabète était considéré comme facteurs de risque dans une analyse multivariée ainsi qu’une durée prolongée de l’intervention [27]. Dans les pays en voie du développement, les fardeaux d’insuffisances en équipement technologique dans les blocs opératoires et l’insuffisance des personnels soignants spécialisés pour les soins chirurgicaux pourraient y contribuer au prolongement de la durée des interventions [20]. De plus, le recours avec retard à la médecine moderne après un état de santé grabataire par certains patients augmenterais l’incidence des ISO par complication ou contamination évolutive. La durée de séjour prolongé à l’hôpital avant l’intervention n’est pas à négliger [10]. En effet, Elle constitue un facteur de risque préopératoire très important, en raison de la modification de la flore microbienne cutanée et digestive dès le 3-4ème jour d’hospitalisation; la fréquence croissante des complications de décubitus (infection urinaire, pulmonaire, cutanée…); la fréquence des explorations invasives et des traitements durant cette période, eux-mêmes responsables d´infections [28]. Beaucoup d’autres facteurs de risque recensés dans les études sont modifiable et maitrisable tels que: l’alcoolisme, le tabagisme, le défaut de préparation des patients, les pathologies sous-jacentes. Ledéfaut de préparation cutanée des malades à opérer est une pratique récurrente dans ces hôpitaux. Elle devait se faire en utilisant unantiseptique de la même gamme que celle utilisé au bloc opératoire. Bien que le choix d’antiseptique approprié pour la douche préopératoire soit discuté [29, 30], sa réalisation reste fondamentale dans la préparation physique du malade. Toutefois, certains facteurs de risque sont similaires à ceux retrouvés dans d’autre pays hors ASS; Au Maroc, les facteurs de risque des ISO retrouvés dans un Hôpital étaient: Le caractère urgent de l’intervention, l´âge, le score ASA, la classe de contamination d´Altemeier, le type d´intervention et la durée opératoire étaient associés au risque infectieux pour la chirurgie viscérale [22]. Les index NNIS établies par les pays développés devraitêtreréajustés pour appliquer dans les pays en développement [31]. Les stratégies de prévention des ISO doivent être appliquées à tout niveau de structure de santé en Afrique Sub-saharien, allant de la surveillance des antibioprophylaxies, de l’usage des antiseptiques, de la préparation des malades à opérer et de création d’une base des données de collecte des informations sur toute intervention chirurgicales [32–34].

## Conclusion

L’incidence des Infections du Site Opératoire en Afrique Sub-saharien est très élevée dans cette revue, avec une hétérogénéité importante selon les études. Des multiples facteurs de risque de ces infections sont liés aux insuffisances de pratiques des soins adéquats, aux plateaux techniques parfois non satisfaisants, au mode de vie des patients et à l’étatde leurs pathologies avancées. Peu de pays et d’études y ont contribués à la recherche des principaux facteurs de risque des ISO en Afrique-Subsaharien. La connaissance de ces derniers permettrait un bon contrôle et la prévention de ces complications chirurgicales. La mise en place de réseaux de surveillance standardisée des ISO pourrait permettre d’améliorer la prévention de ces infections nosocomiales fréquentes et graves.

### Etat des connaissances actuelles sur le sujet

- Les infections du Site Opératoire sont à l’origine des morbidités et des mortalités élevées en Chirurgie en Afrique Subsaharien;
- Son incidence cumulative est élevée et ses facteurs de risque sont multiples allant des pratiques de soins inadéquat à l’état pathologique des patients avancées;
- En Afrique Subsaharienne, l’insuffisance en personnels qualifiés et équipements dans les hôpitaux, les facteurs socio-économiques sont des réels obstacles.

### Contribution de notre étude à la connaissance

- Cette revue a permis de mettre la lumière sur l’état épidémiologique actuel des Infections du Site Opératoire dans les hôpitaux en Afrique Subsaharien;
- Son incidence et ses multiples facteurs de risque ont été détaillés et sont presque commun aux pays d’Afrique-subsaharien;
- Cette étude montre enfin la nécessité d’encourager les publications sur le sujet afin de mieux cerner le problème et réduire les dépenses en santé.
